# Photocatalytic Activity Induced by Metal Nanoparticles Synthesized by Sustainable Approaches: A Comprehensive Review

**DOI:** 10.3389/fchem.2022.917831

**Published:** 2022-09-02

**Authors:** Prashanth Gopala Krishna, Prabhu Chandra Mishra, Mutthuraju Mahadev Naika, Manoj Gadewar, Prashanth Paduvarahalli Ananthaswamy, Srilatha Rao, Sivadhas Rosejanet Boselin Prabhu, Kalanakoppal Venkatesh Yatish, Holenarasipura Gundurao Nagendra, Mahmoud Moustafa, Mohammed Al-Shehri, Saurabh Kumar Jha, Bharat Lal, Sreeja Mole Stephen Santhakumari

**Affiliations:** ^1^ Department of Chemistry, Sir M. Visvesvaraya Institute of Technology, Affiliated to Visvesvaraya Technological University, Bengaluru, India; ^2^ Department of Biotechnology, School of Engineering & Technology, Sharda University, Greater Noida, India; ^3^ Department of Chemistry, Sai Vidya Institute of Technology, Affiliated to Visvesvaraya Technological University, Bengaluru, India; ^4^ Department of Pharmacology, School of Medical and Allied Sciences, KR Mangalam University, Gurgaon, India; ^5^ Department of Chemistry, PES College of Engineering, Affiliated to Visvesvaraya Technological University, Mandya, India; ^6^ Department of Chemistry, Nitte Meenakshi Institute of Technology, Affiliated to Visvesvaraya Technological University, Bengaluru, India; ^7^ Department of ECE, Surya Engineering College, Mettukadai, India; ^8^ Centre for Nano and Material Sciences, Jain University, Bengaluru, India; ^9^ Department of Bio Technology, Sir M. Visvesvaraya Institute of Technology, Affiliated to Visvesvaraya Technological University, Bengaluru, India; ^10^ Department of Biology, Faculty of Science, King Khalid University, Abha, Saudi Arabia; ^11^ Department of Botany and Microbiology, Faculty of Science, South Valley University, Qena, Egypt; ^12^ Department of Biotechnology, School of Applied and Life Sciences (SALS), Uttaranchal University, Dehradun, India; ^13^ Department of Biotechnology Engineering and Food Technology, Chandigarh University, Mohali, India; ^14^ Department of Pharmaceutics, School of Medical and Allied Sciences, KR Mangalam University, Gurgaon, India; ^15^ Department of ECE, Christu Jyothi Institute of Technology and Science, Janagon, India

**Keywords:** nanoparticles, plant extract, characterization, photocatalyst, green synthesis, hazard remediation

## Abstract

Nanotechnology is a fast-expanding area with a wide range of applications in science, engineering, health, pharmacy, and other fields. Among many techniques that are employed toward the production of nanoparticles, synthesis using green technologies is the simplest and environment friendly. Nanoparticles produced from plant extracts have become a very popular subject of study in recent decades due to their diverse advantages such as low-cost synthesis, product stability, and ecofriendly protocols. These merits have prompted the development of nanoparticles from a variety of sources, including bacteria, fungi, algae, proteins, enzymes, etc., allowing for large-scale production with minimal contamination. However, nanoparticles obtained from plant extracts and phytochemicals exhibit greater reduction and stabilization and hence have proven the diversity of properties, like catalyst/photocatalyst, magnetic, antibacterial, cytotoxicity, circulating tumor deoxy ribo nucleic acid (CT-DNA) binding, gas sensing, etc. In the current scenario, nanoparticles can also play a critical role in cleaning wastewater and making it viable for a variety of operations. Nano-sized photocatalysts have a great scope toward the removal of large pollutants like organic dyes, heavy metals, and pesticides in an eco-friendly and sustainable manner from industrial effluents. Thus, in this review article, we discuss the synthesis of several metal nanoparticles using diverse plant extracts, as well as their characterization via techniques like UV–vis (ultraviolet–visible), XRD (X-ray diffraction), SEM (scanning electron microscopy), TEM (transmission electron microscopy), FTIR (Fourier transform infrared spectroscopy), etc., and catalytic activity on various hazardous systems.

## Introduction

Clean water is important for human health and survival. Many countries are currently suffering from a shortage of drinking water. Water scarcity is not only the result of increased population densities, but also due to expansion of several manufacturing sectors such as textiles, automobiles, petrochemical, electronics, food, pharma, etc. (US Environmental Protection Agency, 1998b; World Health Organization, 2008; Herrera et al., 2013). As a result, water reuse has become an important issue in today’s global resource management system. In many developing countries, the industry accounts for about 22% of the world’s total water usage, but more than 70% of untreated industrial waste is simply wastewater (Lin et al., 2007). Dyes typically constitute a major pollutant in waste water. Textile and dye wastes are extremely dangerous to aquatic life and the surrounding environment (Ghorai and Biswas, 2013; Wang et al., 2014a). Such effluents are highly hazardous due to their toxicity, strong colour, high chemical oxygen demand (COD) content, and poor bio-degradability (Jing et al., 2011). Usually, naphthol orange (NO), methylene blue (MB), methyl orange (MO), congo red (CR), acid orange (AO), malachite green (MG), eosin Y (EY), and rhodamine B (RhB) are utilized in a variety of industries, including textile, food, and pharmaceutical. To control the contamination of the ecosystem by such hazardous chemicals, researchers have been exploring cost-effective degradation *via* photocatalytic techniques, which have been widely used over the last two decades as a strategy, especially for dye decomposition in wastewater and effluents (Binding and Steinbach, 1970; Daneshvar et al., 2005; Zhang et al., 2011; Wang et al., 2012; Zhang et al., 2012).

Nanoparticles (NPs) are a class of materials with properties distinctively different from their bulk and molecular counterparts. NPs are considered to be the building blocks for nanotechnology, and are referred to particles with at least one dimension less than 100 nm. Nanomaterials and nanotechnologies have attracted tremendous attention in recent years, due to their effective applications, ranging from traditional chemical to medicinal and environmental technologies (Sevilla et al., 2013; Shume et al., 2020). NPs with dimensions ranging from 1 to 100 nm that exhibit excellent qualities in terms of huge ratio of surface and volume, surface morphology and size (Afsharian and Khosravi-Daran, 2019; Fagier, 2021), have been used by several industries and humankind for thousands of years (Biswas and Wu, 2005). NPs surfaces themselves may be distinctive (Banfield and Zhang, 2001). However, there has been a recent resurgence because of the ability to synthesize on large scales and characterize with accuracies, so as to manipulate their surface properties *via* the particle’s atomic, electronic, and magnetic structures, physical and chemical natures, and reactivity relative to the bulk material (Cho et al., 2013).

A nanoparticle can be either a zero dimensional where the length, breadth and height is fixed at a single point as in nano dots; one dimensional where it can possess only one parameter like in graphene; two dimensional where it has length and breadth, as seen in carbon nanotubes; or three dimensional where it has all the parameters such as length, breadth and height as in gold NPs (Anu Mary Ealias and Saravanakumar, 2017). Hence, NPs are a class of materials with conspicuous properties distinctively different from their bulk and molecular counterparts. Their distinct functional qualities make them a viable candidate for a variety of applications for photocatalysis, catalysis, sensor technology, magnetic property, DNA binding, food, engineering, medical, disease diagnosis and treatment, anti-carcinogenic, antioxidant, anti-tubercular, and antimicrobial (Li et al., 2011; Bera and Belhaj, 2016; Sabouri et al., 2019; Sapawe et al., 2019; Ghosh et al., 2020a; Pillai et al., 2020; Abdullahi Ari et al., 2021; Chandraker et al., 2021a).

Organic compounds discovered as contaminants in industrial or domestic wastewater effluents must be eliminated or destroyed before being discharged into the environment (Prashanth et al., 2022a). The presence of such pollutants in ground and surface water necessitates treatment in order to reach acceptable drinking water quality. The rising public concern about these environmental contaminants has necessitated the development of novel treatment approaches, with photocatalysis gaining a lot of interest in the pollutant degradation sector. An ideal photocatalyst should be stable, inexpensive, non-toxic, and, of course, highly photoactive.

Interestingly, recent literature do not present the complete picture of photocatalytic activity of metal NPs synthesized with the aid of plant extracts, and hence, this review paper tries to address the gaps, by providing insights into the development of novel NPs towards treatment of dye-contaminated wastewater with low price and high efficiency, via photocatalysis.

### Why Green Approach for NPs Synthesis?

NPs are synthesized by two methods: 1) top-down approach and 2) bottom-up approach. In top-down method, bulk materials are transformed to NPs through sputtering technique, grinding, and milling. In bottom-up method, NPs are synthesized through physical, chemical, and biological methods. Different physical and chemical methods include electrochemical changes, chemical reduction, spray pyrolysis, sonication, arc discharge, pulse wire discharge, pulsed laser ablation, radiation, electro-deposition, evaporation–condensation, vapor and gas phase, ball milling, lithography, photochemical reduction, sol–gel, pyrolysis, and combustion, etc. Microwave, precipitation, hydrothermal, combustion, and sonochemical methods are commonly employed for the preparation and stabilization of metallic NPs (Chen et al., 2001; Geethalakshmi and Sarada, 2012; Jamkhande et al., 2019, Krishna et al., 2016a; Prashanth et al., 2016b; Krishna et al., 2017a; Krishna et al., 2017b; Sathyananda et al., 2021a).

Since processes such as kinetics of the metal ion interaction with a reducing agent, the adsorption process of a stabilizing agent with metal NPs, and diverse experimental methodologies have a substantial influence on the shape of metallic NPs; the method of synthesis of NPs is very crucial (Vijayakumar et al., 2013; Ahmed et al., 2022).

There are several disadvantages of chemical methods like they are being toxic, harmful for society, high cost, etc. Chemical methods for the synthesis of metal NPs have been identified as toxic for the environment as well as the synthesis involves high temperature and pressure (Irshad et al., 2021). Further, biomedical applications of these NPs could be toxic (Thirumurugan et al., 2010; Herizchi et al., 2016; Muddapur et al., 2022).

Green synthesis is a subset of green chemistry, born after recognizing the need for sustainable processes in the chemical industry, with the goal of developing safer chemical products and processes that reduce or eliminate the generation and use of hazardous elements. Therefore, there are two goals for green synthesis of nanomaterials: 1) Creating nanomaterials to solve environmental problems, by preventing damage from known pollutants, or by incorporating nanomaterials into environmental technologies to clean up contaminated environments (Ahmed et al., 2022) and 2) minimize damage to human health and environment due to human activities (Sivaraj et al., 2015).

The biological approach of synthesis of NPs follows green chemistry because it is free of toxic substances, clean, and energy-efficient. Biological synthesis derives the creation of the basic principles of green chemistry such as prevention, less hazardous chemical syntheses, use of renewable feedstock, reductive derivatives, etc. As far as the biological approach is concerned, NPs are synthesized using fungi, bacteria, plant, animal sources, etc. The synthesis of NPs from plant extracts has more advantages than other biological sources due to the expensive maintenance of cultures and microbial isolation (Chandraker et al., 2019). Plant extract is commonly used as a crucial component for the NPs synthesis due to its safety and feasibility. Proteins, carbohydrates, enzymes, phenolic acids, and alkaloids are found in plants and are used in the reduction and stabilization processes. Plants are biological substrates that are rich in numerous critical phytochemicals, and hence decrease the need for chemicals as reducing, capping, and stabilising agents during the production of metal NPs from their respective precursor solutions (Singh, 2022).

Furthermore, because leaves contain a lot of metabolites, green synthesis usually uses leaf extracts and this route is safe, bio-compatible, and environment friendly (Thirumurugan et al., 2010; Irshad et al., 2021).

**FIGURE 1:**
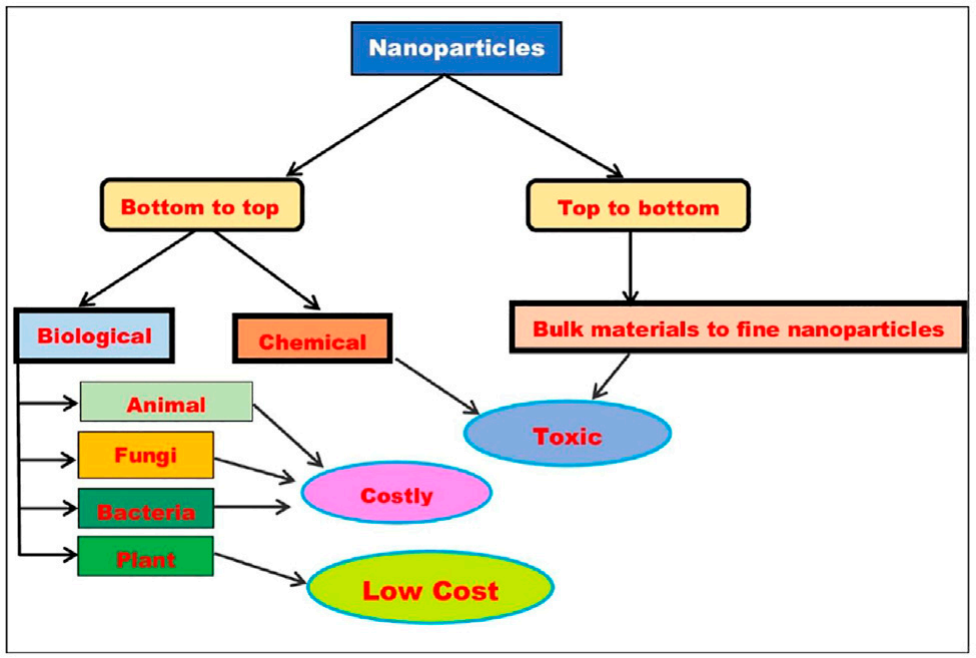
Synthesis process of NPs.

Synthesis process of NPs by various approaches is depicted in Figure 1. In this review article, we highlight the green synthesis process of various metal NPs and their characterization by different techniques such as XRD, FTIR, SEM, TEM, and UV–vis spectroscopy. Apart from this, we highlight their photocatalytic property against different organic pollutant dyes such as NO, MB, MO, CR, RhB, MG, EY, acid orange (AO), and the possible mechanism involved. We believe this review article will be helpful to the new researchers in this field to understand the phenomenon of metal NPs synthesis by green approaches and their photocatalytic activity.

## Synthesis Process of NPs by Plant Extracts

### Preparation of Plant Extract

In this process, leaves collection from plants is a crucial step. Herbal plants are identified by a subject expert before they are collected. Fresh leaves are cleaned with running water followed by double distilled water (DDW), chopped into very small pieces, and dried in shade or in the presence of sunshine. To prepare the extraction, usually a known weight of the powdered leaves (5, 10 g) is soaked in a known volume of DDW (100 mL) and incubated at 40–50°C for 80–100 min. Further, the leaf extract is stored at ambient temperature for cooling before being filtered using Whatman (No. 41) filter paper.

### Phytochemical Tests

Plants are ubiquitous in nature, and many of them have yet to be fully studied for their great health benefits. Plants contain a wealth of essential phytochemicals with therapeutic and medical capabilities (Rizwana et al., 2022).

The medicinal properties of various medicinal plants are due to the phytochemicals contained in them. These phytochemicals are the primary source for treating devastating illnesses. Various phytochemicals have a wide range of activities that help strengthen the immune system and provide long-term disease resistance to protect the body from harmful pathogens (Khalid et al., 2018).

Phytochemical test is the more important aspect in the NPs synthesis from plant extracts. Phytochemical tests reveal the chemical presence in plant extracts. These tests should be performed by the standard protocol (Harbone, 1999; Kokate, 2000; Tiwari et al., 2011; Prashanth and Krishnaiah, 2014a; Prashanth et al., 2015a; Prashanth and Krishnaiah, 2014b). Table 1 provides the different phytochemical tests that are performed for any plant extract. The + ve and -ve signs indicate the presence and absence of the phytochemical of the plant extract, respectively. To examine and investigate the constituents present, phytochemical tests require to be performed.

**TABLE 1:** Phytochemical tests of plant extracts.

Sl. no.	Type of phytochemical	Phytochemical test	Result
1	Anthraquinone	Borntrager’s test	+/−
2	Flavonoids	Alkaline reagent test, Lead acetate test	+/−
3	Anthocyanins	HCl test	+/−
4	Tannins	Lead acetate test	+/−
5	Phytosterols	Libermann-Burchard’s test, Salkowski’s test	+/−
6	Phlobatannins	HCl test	+/−
7	Alkaloids	Mayer’s reagent test, Wagner’s reagent test, Hager's reagent test	+/−
8	Phenol	Killer-Killiani test, Ferric chloride test	+/−
9	Glycosides	Legal's test, Borntrager's test	+/−
10	Terpenoids	Salkowski's test	+/−
11	Saponins	Froth test	+/−
12	Steroids	Burchard's test	+/−
13	Carbohydrates	Molisch's test, Barfoed’s test	+/−
14	Reducing sugars	Fehling's test, Benedict’s test	+/−-
15	Tannins and phenolic compounds	Lead acetate test, Killer-Killiani test, Ferric chloride test	+/−
16	Proteins and amino acids	Ninhydin test, Biuret test	+/−

### Synthesis of NPs

The nature and concentration of the plant extract, pH, temperature, metal salt concentration, and contact time are known to affect the rate of NPs production (Christopher et al., 2015; Ekennia et al., 2022).

**FIGURE 2:**
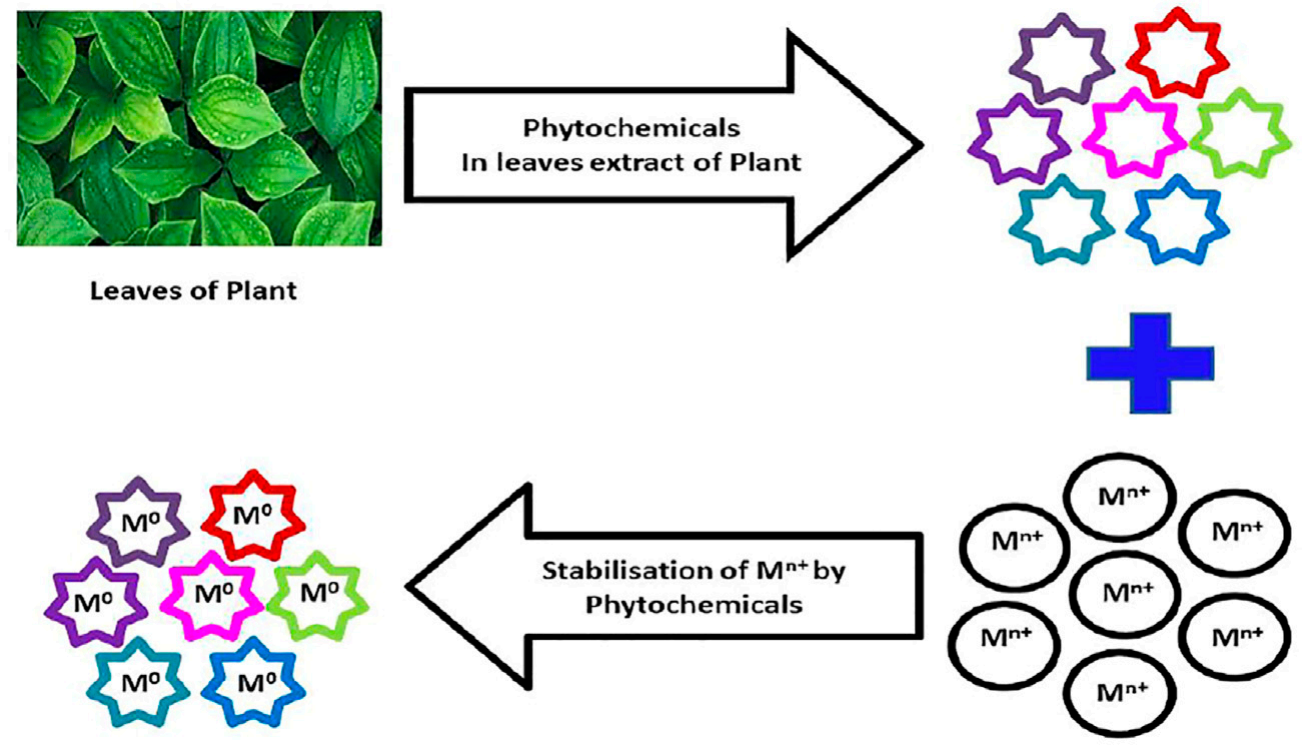
Possible mechanism of synthesis of NPs from leaves extract of plants.

Proper amount of metal salt is dissolved in DDW, and appropriate amount of plant extract is added to it. The mixture is stirred for the accurate time, may be at a normal temperature or at 40–80^°^C. The synthesized NPs are initially observed by visual color change and detected by UV–vis spectroscopy. Finally, the formation of NPs is confirmed by other characterization techniques such as XRD, IR, SEM, and TEM. Plant extracts contain several phytochemicals like anthraquinone, flavonoids, anthocyanine, tannins, phytosterols, phlobatannins, alkaloids, phenols, glycosides, terpenoids, saponins, carbohydrates, and steroids. The phytochemicals from the leaves extract of the plants work as stabilizing and capping agents for synthesizing NPs and in the reduction of M^n+^ to M^o^, as shown in Figure 2. Therefore, capping, stabilizing, and reducing agents are not required for the synthesis of NPs from plant extracts. This is one of the most important advantages of green synthesis of NPs.

### Few Reported NPs Synthesis From Plant Extracts

Sandip Kumar Chandraker et al. synthesized silver (Ag) NPs by using *Justicia adhatoda* (JA) leaf extract. They initially confirmed the formation of JA@Ag NPs by UV–vis spectroscopy and finally confirmed the same by XRD, IR, SEM, and TEM analyses. The nano-size of JA@Ag NPs was 19 nm, which was supported by SEM and TEM analyses. The spherical shape NPs had a semiconducting property with an optical band gap of 3.3 eV (Chandraker et al., 2021b).

**FIGURE 3:**
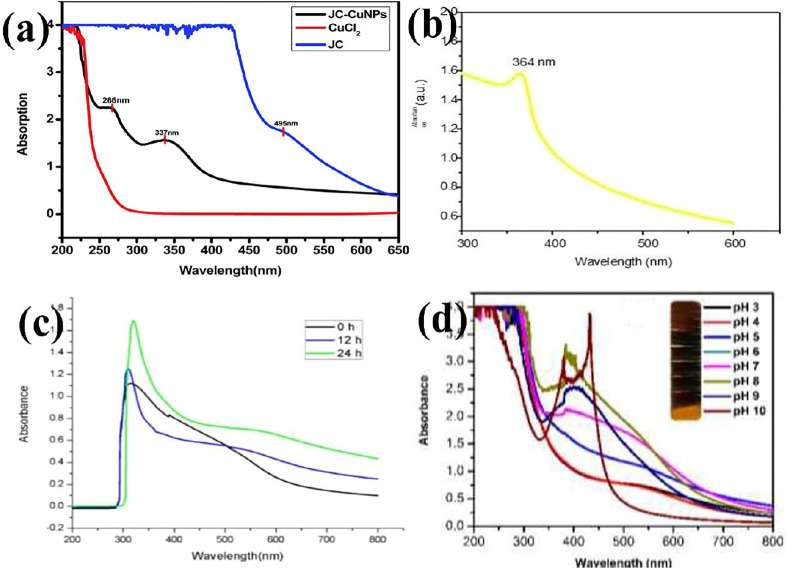
UV–vis absorption spectra of **(A)** JC-Cu NPs (Ghosh et al., 2020b), **(B)** ZnO NPs (Singh et al., 2019a), **(C)** Se NPs at different time intervals (0, 12, and 24 h) (Alagesan and Venugopal, 2019), and **(D)** optimizing parameters to produce Ag_2_O NPs with different pH values by UV–vis spectroscopy (Manikandan et al., 2017).

Ghosh et al. synthesized copper (Cu) NPs by using the leaf extract of *Jatropha curcas* (JC). The JC-Cu NPs were characterized by XRD, UV–vis spectroscopy, FT-IR, SEM, and TEM (Ghosh et al., 2020b). The average crystallite size was 12 ± 1 nm. The JC-Cu NPs exhibited several applications like photocatalysis, semiconductor property, and CT-DNA binding (Ghosh et al., 2020b). Their band gap was found to be 3.6 eV at 337 nm, as shown in Figure 3A (blue: plant extract (JC); red: cuprous chloride; and black: JC-Cu NPs (Ghosh et al., 2020b). Jagpreet Singh et al. synthesized zinc oxide (ZnO) NPs, making use of aqueous leaves extract of *Coriandum sativum*. A sharp absorption peak observed at 364 nm confirmed the formation of zinc oxide (ZnO) NPs, as shown in Figure 3B. The optical band gap determined from absorption spectra was 3.4 eV, which is higher than that of bulk ZnO (3.32 eV). These NPs were employed for the photocatalytic degradation of yellow 186 dye under direct sunlight (Singh et al., 2019a).

Selenium (Se) NPs were synthesized by Venkatesan Alagesan and Sujatha Venugopal with the aid of aqueous leaves extract of *Withania somnifera* (Alagesan and Venugopal, 2019) The UV–vis spectrum of Se NPs at different time intervals (0, 12, and 24 h) is given in Figure 3C. The synthesized Se NPs were tested for the antioxidant activity, antibacterial activity against pathogenic bacterial strains, *Bacillus subtilis, Escherichia coli, Klebsiella pneumoniae*, and *Staphylococcus aureus*, antiproliferative activity, and photocatalytic activity on MB under sunlight. The results suggested that these NPs effectively degraded MB under sunlight irradiation.

Silver oxide (Ag_2_O) NPs were synthesized using *Artocarpus heterophyllus* rind extract, and their antifungal activity was evaluated. Their UV–vis absorption spectra are presented in Figure 3D (Manikandan et al., 2017).

Jyoti et al. synthesized Ag NPs through 10^–3^ M of 95% silver nitrate (AgNO_3_) and 5% leaves extract of Zanthoxylum armatum at 40°C. They initially monitored the formation of NPs by visual color change from brownish to colorless, which was confirmed by UV–vis spectroscopy. Finally, the formation of NPs was asserted by XRD, TEM, SAED, and SEM–EDX (energy-dispersive X-ray spectroscopy). Sizes of the NPs were found to be from 10 to 50 nm. The NPs had a potential photocatalytic activity against organic pollutant dyes (Jyoti and Singh, 2016).

ZnO NPs were synthesized by the solution combustion method with the aid of *Abutilon indicum (AI)*, *Melia azedarach (MA)*, and *Indigofera tinctoria (IT)* leaves extracts with their different concentrations. The average crystallite sizes of the samples were found to be 7, 11, and 12 nm (with different volumes of AI), 7, 9, and 18 nm (with different volumes of MA), and 8, 11, and 12 nm (with different volumes of IT). The samples were characterized by XRD, SEM, TEM, X-ray photoelectron spectroscopy, thermogravimetric analysis, and Brunauer–Emmett–Teller (BET). They were found to exhibit anticancer activity against DU-145 and Calu-6 cancer cell lines (Prashanth et al., 2018).

Lemon juice was employed as a reductant in synthesizing ZnO NPs (Gopala Krishna et al., 2017; Prashanth et al., 2019; Prashanth et al., 2020). The samples were characterized by XRD, SEM with EDS (energy-dispersive spectroscopy) and elemental mapping, TEM, and BET. The samples were tested against DU-145, Calu-6, PC-3, HCT116, A549, MDA-MB-231, and MCF-7 cell lines. The green synthesized ZnO NPs were found to possess antitubercular activity against *Mycobacterium tuberculosis* H37 Ra. They were also found to possess antibacterial activity against different organisms.

ZnO NPs were synthesized from the aqueous fruit extracts of *Punica granatum* and *Tamarindus indica* (Prashanth et al., 2015b). The samples were found to have crystallite sizes ranging from 4-20 nm. They were tested against bacterial strains and MCF-7 cell line.

Synthesis of cobalt (Co)-doped copper oxide (CuO) NPs was carried out using lemon juice as a reductant, and the samples were characterized by XRD and SEM with EDS and elemental mapping. They were tested against *Mycobacterium tuberculosis, H37Rv, Mycobacterium abscessus, Mycobacterium fortuitum*, *Mycobacterium chelonae,* and anticancer activity on MDA-MB-231 (Sathyananda et al., 2021b).

ZnO NPs were synthesized from *Syzgium cumini* (*S. cumini*). FTIR spectroscopy analysis ascertained the existence of flavonoids, phenolic acids, enzymes, and steroids in leaves extract of *S. cumini* which aided in reducing zinc salt into zinc ions and acted as capping agents during the synthesis of NPs. The NPs were tested for seed germination and used as a catalyst for the degradation of RhB (Rafique et al., 2022).

Prashanth et al. reported the synthesis of Ag/CuO nanocomposites by solution combustion method using lemon juice as a reductant (Prashanth et al., 2022b). The samples were subjected to investigation for their antimycobacterial activity on *Mycobacterium tuberculosis H37Rv, Mycobacterium abscessus, Mycobacterium fortuitum, Mycobacterium chelonae*, anticarcinogenic activity on breast cancer cell line MDA-MB-231, and scavenging activity by the 2, 2-diphenyl-1-picrylhydrazyl hydrate (DPPH) method (Prashanth et al., 2022b).

**FIGURE 4:**
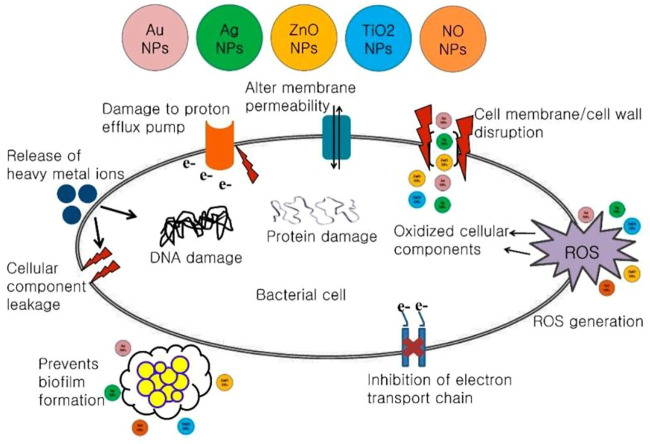
Probable antimicrobial mechanism of different metal NPs (Shaikh et al., 2019).

Several mechanisms have been proposed to explain the antimicrobial mechanism of different metal NPs: inhibition of cell wall/membrane synthesis, disruption of energy transduction, production of toxic reactive oxygen species (ROS), photocatalysis, enzyme inhibition, and reduced DNA production (Weir et al., 2008; Shaikh et al., 2019). The probable antimicrobial mechanism of different metal NPs as explained by Shaikh et al. is shown in Figure 4 (Shaikh et al., 2019).

**FIGURE 5:**
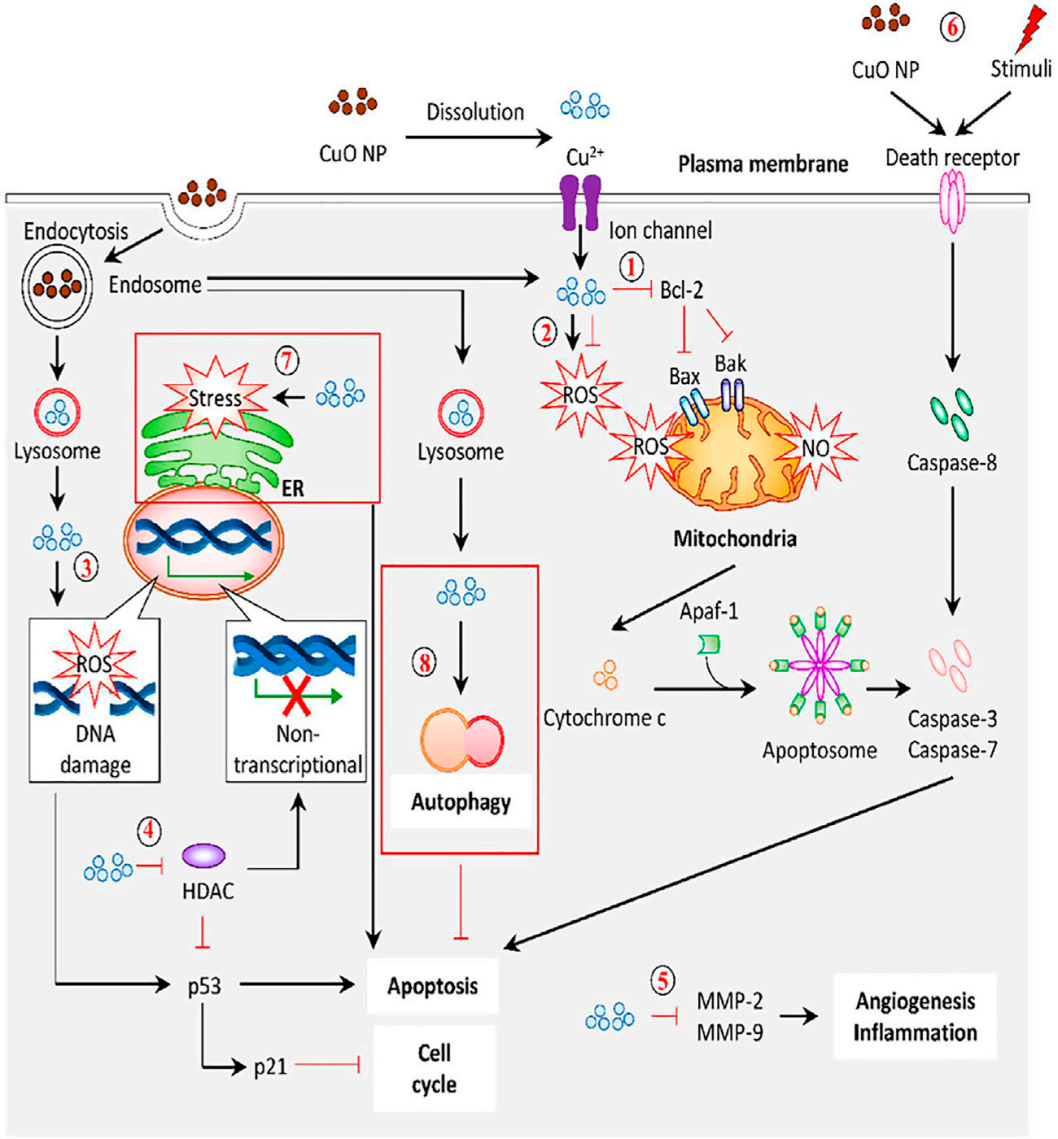
Proposed anticancer mechanism of metal oxide NPs (Letchumanan et al., 2021).

The anticancer potential of these NPs was attributed to the production of ROS in cellular compartments that eventually leads to the activation of autophagic, apoptotic, and necrotic death pathways (Andleeb et al., 2021). The plausible anticancer mechanism of CuO NPs proposed by Letchumanan et al. is presented in Figure 5 (Letchumanan et al., 2021).

In summary, during the last decade, many efforts have been made in developing variety of metal NPs by green routes, making use of plant materials as reducing/stabilizing agents. Leaves, root, bark, stem, and fruits of various plants have been employed to develop different metal NPs, which possess multiple applications. The distinctive features of NPs synthesized using plant extracts enhance their applications in many fields. Efforts need to be intensified in manipulating the morphological characteristics of NPs by means of various parameters such as the nature of the plant materials, reaction time, reactant concentrations, pH, and temperature. Such parameters are very important to understand their crucial roles and effects during the synthesis of NPs.

## Characterization

Any scientific research work depends on the characterization of the samples. The formation of NPs, their size, morphology, and surface area are determined from the instrumental characterization. NPs are characterized by different techniques like FTIR, UV–vis spectroscopy, SEM, TEM, energy-dispersive X-ray (EDX), and XRD. The UV–vis spectra confirms the formation of NPs by comparison peaks of metal salt and leaves extract. The XRD analysis is of elucidating the crystal parameter, structure, and size. The SEM analysis is for measuring the surface morphology of the NPs and size. The TEM determines the exact size of the synthesized NPs. The EDX defines the percentage of elements present in the NPs. UV–vis spectroscopy, FTIR spectroscopy, TEM, and SEM are among the few that are the commonly used techniques for NPs characterization (Chandraker et al., 2021c).

### XRD Analysis

**FIGURE 6:**
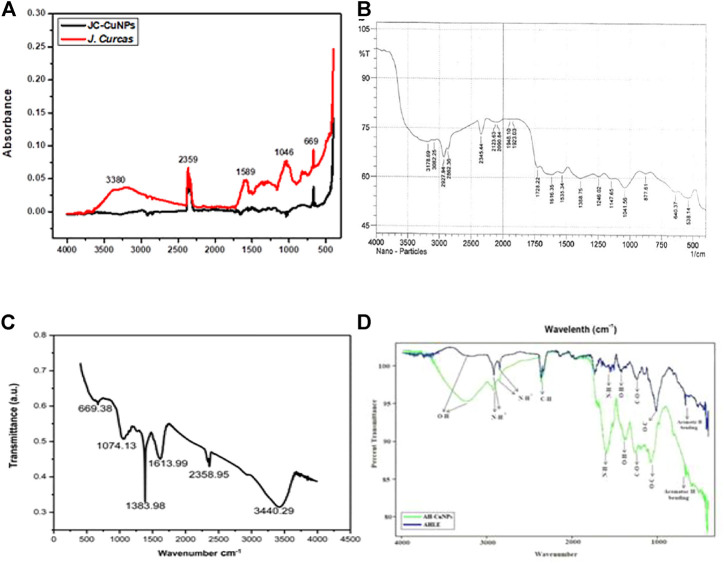
FT-IR spectra of **(A)** JC-Cu NPs (Ghosh et al., 2020b), **(B)** CO-Ag NPs (Zia et al., 2016), **(C)** AC-Ag NPs (Chandraker et al., 2019), and **(D)** AH-Cu NPs (Chandraker et al., 2020).

Ghosh et al. characterized the JC-Cu NPs by XRD as shown in Figure 6A (Ghosh et al., 2020b). They calculated the nano-size from XRD, and it was found to be 12 ± 1 nm. Figures 6B–D describe the XRD analysis of Ag NP synthesized using *Cydonia oblong* seed extract (CO-Ag NPs) (Zia et al., 2016), *Ageratum conyzoides* leaf extract (AC-Ag NPs) (Chandraker et al., 2019) and *Ageratum houstonianum* leaf extract (AH-Cu NPs) (Chandraker et al., 2020) respectively. The crystallite sizes of CO-Ag NPs and AC-Ag NPs were 38 and 35 nm, respectively.

### FT-IR

**FIGURE 7:**
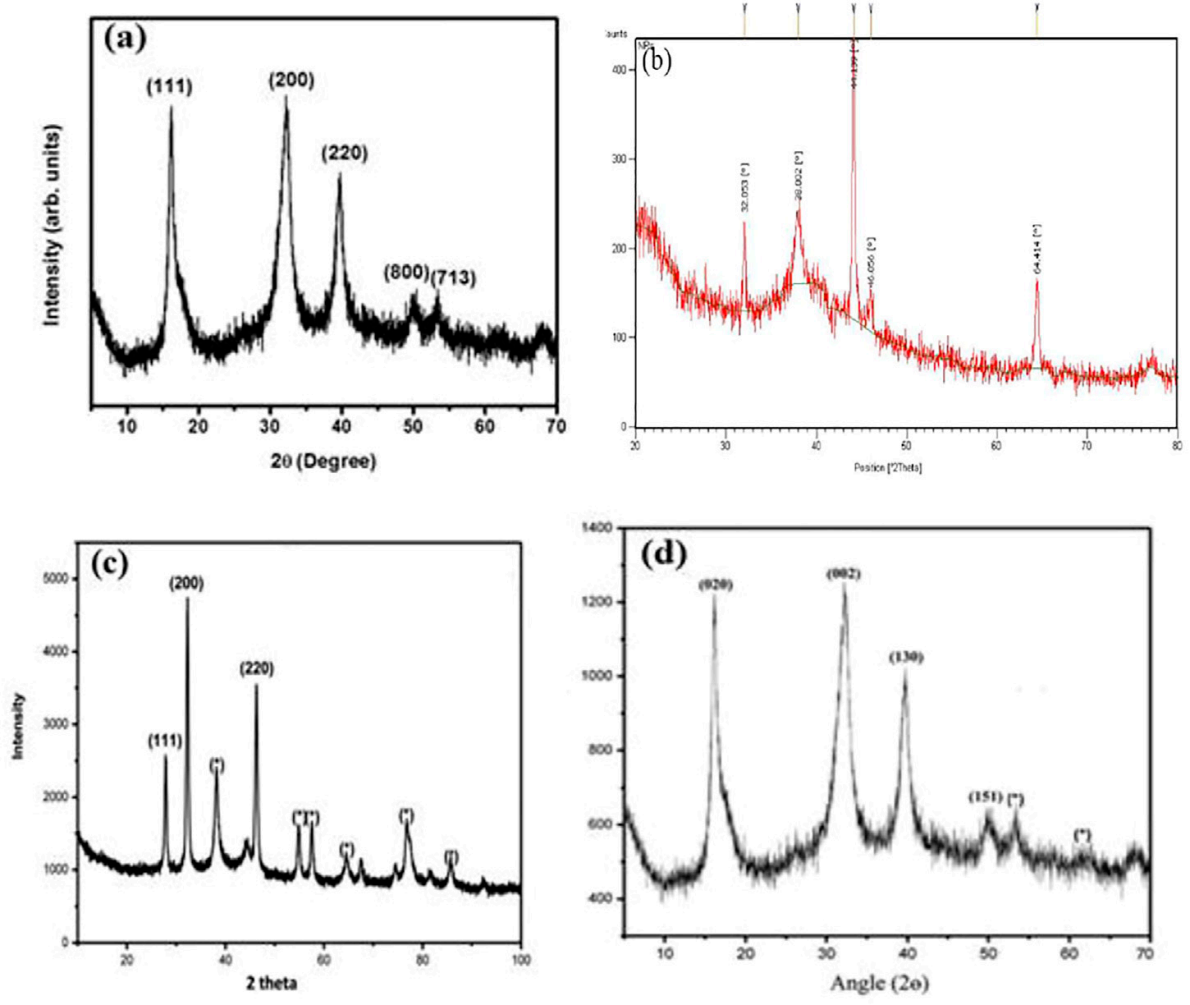
XRD analysis of **(A)** JC-Cu NPs (Ghosh et al., 2020b), **(B)** CO-Ag NPs (Zia et al., 2016), **(C)** AC-Ag NPs (Chandraker et al., 2019), and **(D)** AH-Cu NPs (Chandraker et al., 2020).

The absorption bands for JC-Cu NPs at 418, 669, 1046, 1589, 2359, and 3380 cm^−1^ were due to C-Cl, C-O, C=N, C-H, and O-H functional groups, as depicted in Figure 7A (Ghosh et al., 2020b). The functional groups in JC might be accountable for the bio-reduction of Cu^+^ to JC-Cu NPs (Ghosh et al., 2020b). The peak for the NH stretching was observed at 3178.60 cm^−1^. The peaks at 3082.25 and 2862.36 cm^−1^ were associated with the symmetric CH_2_ stretching (Zia et al., 2016). The peak at 2927.94 cm^−1^ was associated with the CH_2_ asymmetric stretching. The peak at 2123.63 cm^−1^ indicated the C:C stretching and that at 1948.10 cm^−1^ was associated with allenes (C:C:C). The peak for the carbonyl group was obtained at 1728.22 cm^−1^. The carbonyl groups proved the presence of flavanones or terpenoids that are adsorbed on the surface of the metal nano-sized particles by interaction through p-electrons in the carbonyl groups in the absence of a sufficient concentration of chelating agents [Figure 7B (Zia et al., 2016)**]**.

The IR-spectrum at 1074.83, 1383.98, 1613.99, 2358.95, and 3440.29 cm^−1^ were identified as C-OH, alcohol/ethers/esters/carboxylic acids/amino acids, C=O, C-H, N-H stretching respectively, as shown in Figure 7C (Chandraker et al., 2019).

The IR-spectra at 3236.34, 2918.71, 2359.93, 1595.13, 1377.76, 1260.17, 1074.64, and 667.81 cm^−1^ were identified as the O–H stretch, N^+^-H, C-H, N-H, O-H, C-O, O-C, and aromatic H respectively [Figure 7D (Chandraker et al., 2020)**]**.

### SEM

**FIGURE 8:**
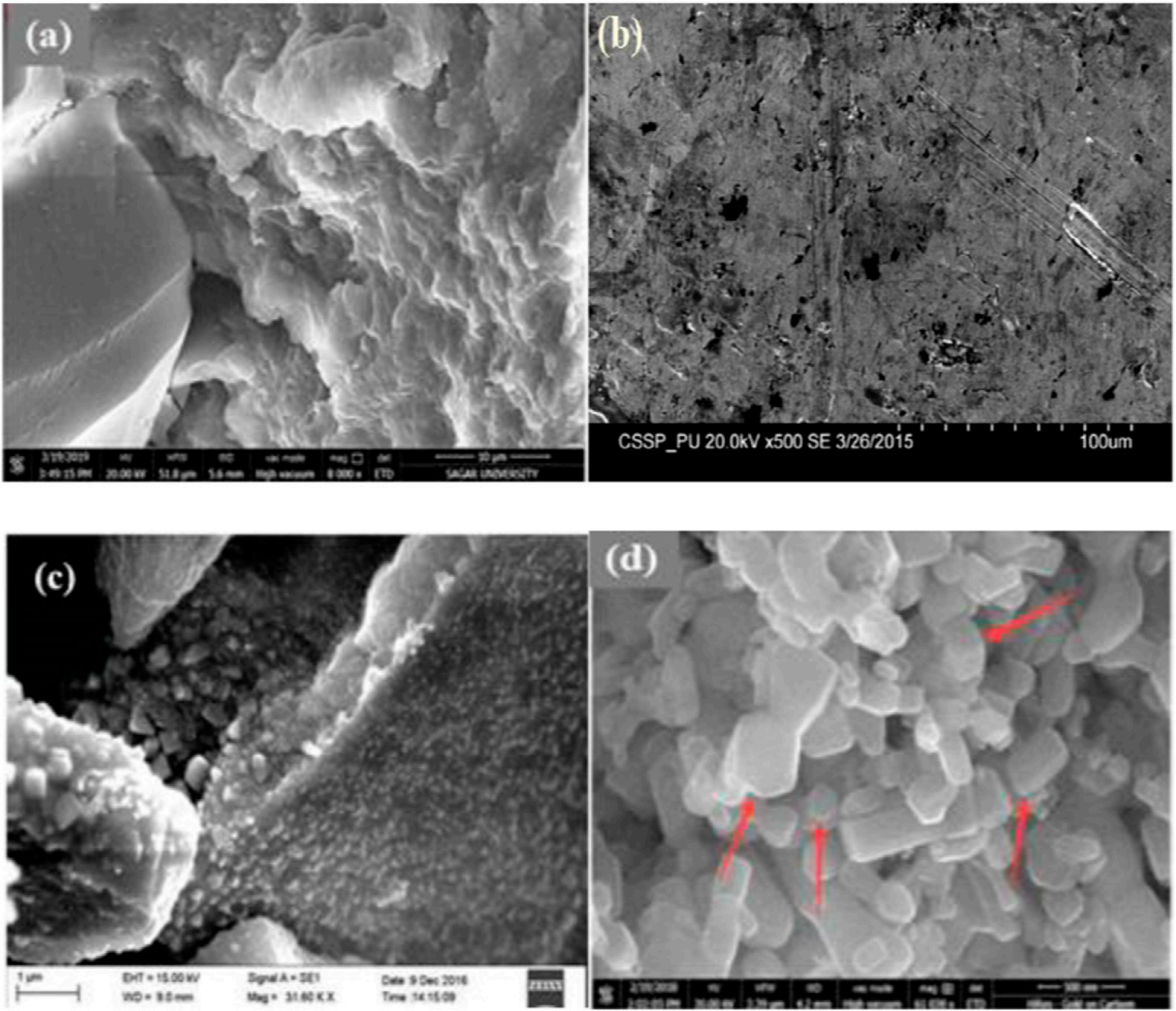
SEM analysis of **(A)** JC-Cu NPs (Ghosh et al., 2020b), **(B)** CO-Ag NPs (Zia et al., 2016), **(C)** AC-Ag NPs (Chandraker et al., 2019), and **(D)** AH-Cu NPs (Chandraker et al., 2020).

The morphology and shape of NPs are described by the SEM images. Few SEM images of NPs obtained from plant extracts are presented in Figures 8A–D. SEM images showed that *Cydonia oblong* (CO)-Ag NPs have been formed and Ag^+^ ions have been completely consumed, as presented in Figure 8B (Chandraker et al., 2019). The shapes of AH-Cu NPs were hexagonal, rectangular, and cubic (Figure 8D) (Chandraker et al., 2020).

## Photocatalytic Activity

### Optical Band Gap

The optical band gap of NPs is a critical factor in photocatalytic activity. The optical band gap may predict the photocatalytic activity of the NPs. When the band gap value is less than 5 eV, it favors the photocatalytic activity of the NPs. The optical band gap is calculated from the UV–vis spectrum of the NPs. It is calculated by using Tauc’s formula, as shown in Eq. 1 (Ghosh et al., 2020b):

$${\left(\alpha h\upsilon \right)}^{n}=A\left(h\upsilon -E_{g}\right)$$ (1)

where *α* is the absorption coefficient, *h* is Plank’s constant, *υ* is the frequency, *A* is a constant, *E*_*g*_ is the band gap, and n signifies the exponent value.

**FIGURE 9:**
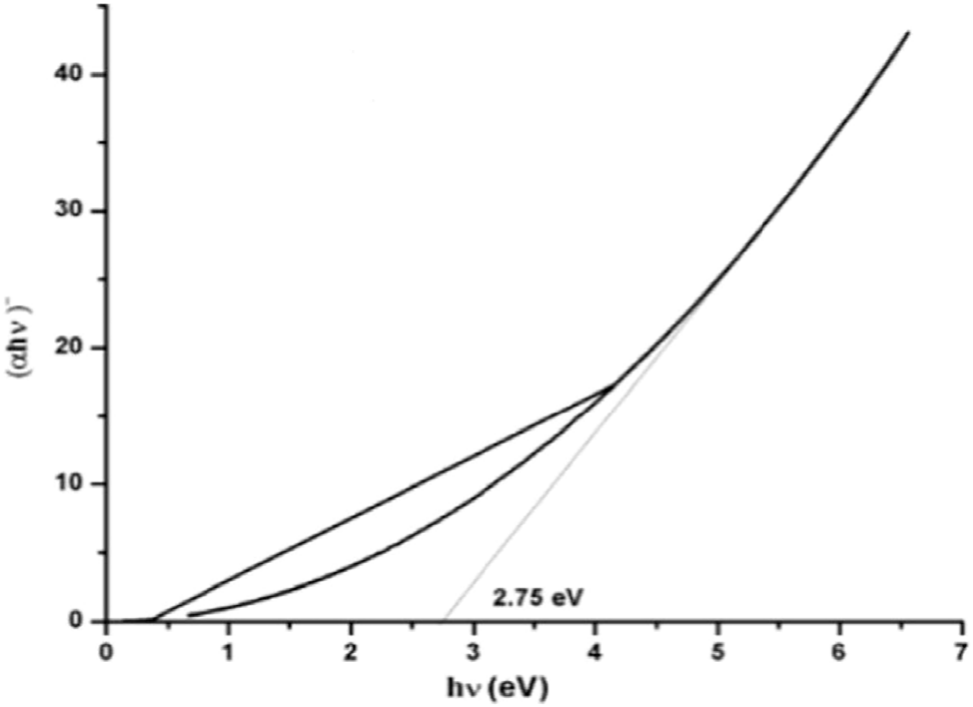
Band gap energy of Se NPs at 24 h (Alagesan and Venugopal, 2019).

Ghosh et al. reported that the optical band of JC-CuNPs was 3.6 eV and it was very active in photocatalysis (Ghosh et al., 2020b). Chandraker et al. reported a band gap value of 3.33 eV for Ag NPs synthesized from plant extract (Chandraker et al., 2021c). From the plot (Figure 9), the band gap energy of Se NPs was found to be 2.75 eV (Alagesan and Venugopal, 2019).

### Method of Photocatalytic Reaction by NPs

Photocatalytic tests are carried out by employing NPs, an aqueous solution of organic dye, and in the presence of sunlight. The reactions are carried out by the addition of NPs as a photocatalyst (0.05 g) into each batch of 30 mL aqueous solution of the organic dyes, and then the reaction mixtures are exposed to sunlight. The reaction mixtures are monitored by UV–vis spectroscopy at a constant time interval (Jiang et al., 2015).

### Kinetics of the Reaction

Pseudo-first-order, pseudo-second-order, and Langmuir–Hinshelwood kinetic expressions are widely used to describe the kinetics study of the photocatalytic oxidation of organic compounds. The reaction of kinetics can be evaluated by Eq. 2 (Ahmad and Majid, 2018):

$$\ln\frac{\left[A\right]}{\left[A_{0}\right]}=-kt$$ (2)

where k is the rate constant of pseudo-first-order, [A_0_] is the initial concentration of organic dyes at time t = 0, and [A] is the concentration of organic dyes at time t. The rate constant of the reaction is determined by plotting ln [A]/[A_0_] vs*.* t and slope with rate constant (k).

### Efficiency of Degradation of Dyes by NPs

According to Beer’s law, the concentration of the organic dye is linearly proportional to the intensity of the dye’s absorption peak and the effectiveness of NPs for organic dye degradation, which may be computed as follows (Eq. 3) (Jayappa et al., 2020):

$$\text{Efficiency}\left(\%\right)=\left(1-\frac{A_{t}}{A_{o}}\right)\times 100$$ (3)

where A_t_ is the absorbance at t time and A_0_ is the initial absorbance at zero time.

### The Possible Mechanism

**FIGURE 10:**
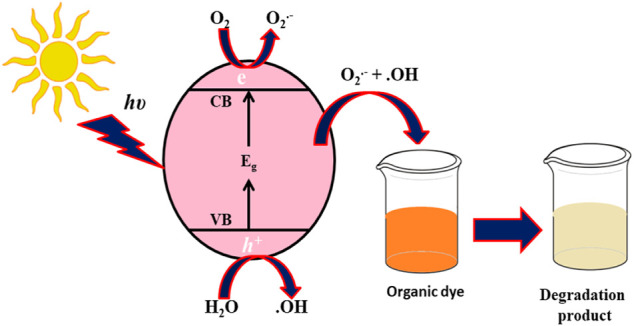
Possible mechanism of dye degradation by NPs.

The first step involves the adsorption of the dye onto the surface of NPs and the generation of electron hole (e^−^/h^+^) in the presence of sunlight (Eq. 4). The electron (e^−^) in conductance band (CB) reacts with oxygen and superoxide anionic radical (O_2_^−^) (Eq. 5). The hole (h^+^) in the valence band (VB) reacts with water molecules and forms a hydrogen ion (H^+^) and hydroxyl anion (OH^−^) (Eq. 6). At the same time, another hole (h^+^) reacts with water molecules and produces hydroxyl radicals (OH) (Eq. 7). Concurrently, dye is activated by the absorption of sunlight and produces dye* (Eq. 8) while instantaneously discharging electrons to form dye^+^ (Eq. 9). The dye^+^ reacts with highly unstable species, hydroxyl radicals (OH^.^) and superoxide anionic radical (O_2_^−^), and produces the degradation products in the form of CO_2_ and H_2_O (Eqs 10, 11) (Lam et al., 2012; Ibhadon and Fitzpatrick, 2013; Ong et al., 2018). The possible mechanism is shown in Figure 10:

$$\text{NPs}+\text{h}\upsilon \to {\text{e}}^{-}\left(\text{CB}\right)+{\text{h}}^{+}\left(\text{VB}\right)$$ (4)

$${\text{O}}_{2}+{\text{e}}^{-}\to {\text{O}}_{2}^{\cdot -}$$ (5)

$${\text{H}}_{2}\text{O}+{\text{h}}^{+}\to {\text{H}}^{+}+{\text{OH}}^{-}$$ (6)

h++H2O→O⋅H (7)

Dye+hυ→Dye∗ (8)

$$\text{Dye}*\to {\text{Dye}}^{+}+\text{e}$$ (9)

$${\text{Dye}}^{+}+{\text{OH}}^{\cdot }\to \text{Degradation}\;\text{products}$$ (10)

$${\text{Dye}}^{+}+{\text{O}}_{2}^{\cdot -}\to \text{Degradation}\;\text{products}$$ (11)

### Some Case Studies of Photocatalytic Activity of Green Synthesized NPs

Cu NPs were synthesized by the green method. The optical band gap (4.5 eV) favored the photocatalytic activity. CR was degraded by AH-CuNPs, and the rate of reaction was 3.1 × 10^–4^ s^−1^ (Chandraker et al., 2020).

CuO NPs were synthesized by using plant extract of *Ruellia tuberosa* and *Abutilon indicum*. CuO NPs exhibited photocatalytic activity against Crystal Violet (CV) and Acid Black 210 (Ijaz et al., 2017; Vasantharaj et al., 2019a).

Biogenic synthesis of CuO NPs using *Psidium guajava* leaf extract as a reducing agent has been reported (Singh et al., 2019b). The CuO NPs exhibited excellent degradation efficiency for the industrial dyes, i.e., Nile blue (NB) (93% removal in 120 min) and reactive yellow 160 (RY160) (81% removal in 120 min) with apparent rate constants of 0.023 and 0.014 min^−1^, respectively. The CuO catalyst was found to be reusable for photocatalytic dye degradation even after five consecutive cycles.

Biocompatible CuO NPs were produced with the aid of *Rubia cordifolia* bark extract (Vinothkanna et al., 2022). The photocatalytic activity of the CuO NPs demonstrated the MB (81.84%) and CV (64.0%) dye degradation potentials, indicating the environmental bioremediation efficacy (Vinothkanna et al., 2022).

Phytocombustion method was used to produce CuO NPs, making use of *Pouteria campechiana* leaves extract (Navada et al., 2021). The NPs degraded MB and MO with magnificent degradation more than 84% and its reusability over five cycles owing to its stability (Navada et al., 2021).

CuO and Ag/CuO NPs were synthesized using *Cyperus pangorei* extract by co-precipitation method. The plant extract acted as a reducing and stabilizing agent. The photocatalytic activity of CuO and Ag/CuO NPs was analyzed against RhB under visible light irradiation. The presence of metallic silver on the surface of CuO increased the electron‐hole pair life and improved the effective catalytic activity (Parvathiraja and Shailajha, 2021).

Suresh ChandMali reported the photocatalytic degradation of MB by Cu NPs synthesized by the green approach using *Celastrus* *paniculatus willd* leaf extract. The experiment used a 10 mg L^−1^ concentration of MB mixed with 10 mg dosages of photocatalyst. Almost complete degradation of MB was seen for 120 min. In the presence of Cu NPs, the photodegradation was significantly enhanced at basic pH (pH = 9) (Mali et al., 2020).

MnO_2_, CuO, and Ag NPs were synthesized by *Kalopanax pictus* leaf extract. In this study, manganese dioxide (MnO_2_) NPs had potential photocatalytic activity against CR than the other two NPs (Moon et al., 2018).

SA-Ag NPs were synthesized by using AgNO_3_ and plant extract of *Sonchus arvensis* (SA). SA-Ag NPs had effective photocatalytic activity against MB due to a lower band gap (3.2eV) (Chandraker et al., 2021c; Chandraker et al., 2019).

BP-Ag NPs were formed by using AgNO_3_ and leaf extract of *Bryophyllum pinnatum* (BP). Spherical shaped BP-Ag NPs degraded MB and RhB in the presence of sunlight (Chandraker et al., 2021a).

Ag NPs were prepared by using a green route with extracts of onion, tomato acacia catechu, and their mixed extracts by Kishore Chand et al. (Chand et al., 2020). These Ag NPs were tested for the degradation of MO, MR, and CR room temperature. In the presence of Ag NPs prepared with onion and acacia catechu, MO and MR dyes were completely degraded within a very short reactive time of 20 min, whereas in the case of CR dye, it was 15 min. Ag NPs synthesized with the mixture had increased effect on the degradation of all dyes as compared to Ag NPs synthesized alone in any one extract.

Green synthesis of Ag NPs using the leaf extract of *Kalanchoe Daigremontiana* was carried out, and the photocatalytic activity of the Ag NPs was evaluated through the degradation of MB, where the degradation time as low as 1 min was reported (Molina et al., 2019).

Shaikh et al. have reported the biosynthesis of Ag NPs using *Shorea robusta* leaf extract (Shaikh et al., 2020). The photocatalytic degradation process by the bio-synthesized Ag NPs exhibited a significant degradation potential for RhB (maximum degradation efficiency up to 90.41%) (Shaikh et al., 2020).

Green synthesis of Ag NPs from *procera* plant latex has been reported (Chandhru et al., 2021). The Ag NPs were applied as photocatalysts for the degradation of MO dye and also as anti-biofilm agents. The photocatalytic degradation efficiency was determined to be 0.0076.

Leaves of *Cordia dichotoma* were employed in the biosynthesis of Ag NPs (Dave et al., 2021)*.* The degradation process of MO was completed at 70 h and was recognized by the change of reaction mixture color to colorless.

Rama Sharma et al. reported the synthesis of Ag NPs using *Terminalia bellirica* fruit extract (Sharma, 2021). The photocatalytic degradation of MB by these biosynthesized Ag NPs was evaluated by UV–vis spectroscopy.

Biosynthesis of Ag NPs using *Berberis vulgaris* extract was reported (Hashemi et al., 2022a). The results showed the average crystallite size of 45–60 nm and spherical morphology with only a few agglomerated particles. These NPs exhibited high photocatalytic degradation (97.82%) in short time on MO.

Panneerselvi et al. reported *Mangifera indica* resin-assisted synthesis of Ag NPs (Panneerselvi et al., 2022). The biosynthesized Ag NPs effectively degraded MB.

Green synthesis of Ag NPs was achieved using the *Catharanthus roseus* flower extract as the reducing agent (Muthu et al., 2021). These NPs had excellent photocatalytic activity in the reduction of methyl orange.

Synthesis of Ag NPs using *Sambucus ebulus* phenolic extract has been reported (Hashemi et al., 2022b). The biogenic Ag NPs were used as a nanocatalyst, which showed a high catalytic activity for the degradation of MO under sunlight irradiation.

Phytosynthesis of silver/silver chloride particles (Ag/AgCl) NPs was carried out using *Cissus quadrangularis* stem extract under microwave irradiation (Priyadarshini et al., 2021). Ag/AgCl NPs demonstrated a high photocatalytic activity toward the degradation of environmental pollutant MB under visible light illumination.

Ag/AgCl nanocomposites were obtained from the *Azadirachta indica* plant fruit extract (Panchal et al., 2021). The sample exhibited an excellent dye decomposition efficiency of 92.5% on MB in 80 min, which was better than the performance of Ag NPs and AgCl NPs.

Cadmium sulfide (CdS)-NPs were synthesized by *Dicliptera Roxburghiana* plant extract. It degraded MB in the presence of sunlight, and the efficiency of degradation was 87.12% (Ullah et al., 2021).

Copper–cobalt–nickel (Cu–Co–Ni) NPs were synthesized by *Origanum vulgare* leaves extract. The NPs had a photocatalytic activity against MB, and the order of reaction was pseudo-first-order (Alshehri and Malik, 2020).

Cobalt (Co) NPs were synthesized by *Taraxacum officinale*. Co NPs degraded the MO dye within 60 min in the presence of sunlight (Rasheed et al., 2019a).

*α*-ferric oxide (α-Fe_2_O_3_) NPs were synthesized by using ferric chloride and leaf extract of *Carica papaya*. Remazol yellow RR dye was degraded to 76.6% extent by α-Fe_2_O_3_ NPs in the presence of light (Bhuiyan et al., 2020).

Iron oxide NPs (IO NPs) were synthesized by *Teucrium polium* leaf extract. IO NPs had a photocatalytic activity against MO. The degradation efficiency was 73.6% within 6 hours (Kouhbanani et al., 2019).

Ferric oxide (FO) NPs were synthesized by *Peltophorum pterocarpum* leaf extract. MB was degraded by FO NPs in the presence of sunlight (Anchan et al., 2019).

FO NPs were formed with the aid of the leaf extract of *Ruellia tuberosa*. CV was degraded to 80% by FO NPs in the presence of sunlight (Vasantharaj et al., 2019b).

Iron-polyphenol nanoparticles (Fe-P-NPs) were synthesized by three plant extracts *Melaleuca nesophila*, *Rosemarinus officinalis,* and *Eucalyptus tereticornis*. Fe-P-NPs degraded 80% (AB 194) (Wang et al., 2014b).

Iron oxide (Fe_3_O_4_) NPs developed by different plant extracts (leaves of *Mentha pulegium*, *Artemisia herba-alba*, fruit peels of *Punica granatum*) were proved to possess catalytic activity for the dye degradation of MB and CR stains under environmental conditions. The NPs were proved to be useful in the treatment of wastewater/dye degradation (Meneceur et al., 2022).

Novel iron oxide NPs were produced, making use of *Peltophorum pterocarpum* plant extract (Shah et al., 2022). The as-prepared NPs effectively removed the RhB molecules with a high removal rate of 95.1% under UV irradiation.

Palladium (Pd) NPs were formed by the plant extract of *Pimpinella tirupatiensis*. Pd NPs had a photocatalytic activity against CR (Narasaiah et al., 2017).

ZnO NPs were synthesized by using the plant extract of *Plectranthus amboinicus*. ZnONPs showed photocatalytic activity against MR (Fu and Fu, 2015).

Panous extract-mediated ZnO nanoflowers were synthesized and tested for the removal of MB, eosin Y (EY), and MG (Kaliraj et al., 2019). The nanoflowers degraded the 15 mg L^−1^ MB, EY, and MG to >99% within 80, 90, and 110 min of contact time, respectively. In addition, the ZnO photocatalyst removed the low concentrated 5 mg L^−1^ of MB, EY, and MG within 30, 35, and 40 min of contact time, respectively (Kaliraj et al., 2019).

Aloe vera gel was used in the sol–gel synthesis of tin oxide and zinc oxide (SnO_2_-ZnO) nanocomposites. They were tested for their photocatalytic activity on MO (Sudhaparimala and Vaishnavi, 2016).

ZnO NPs synthesized using the leaf extract of *Cyanometra ramiflora* were tested for the photocatalytic degradation of pollutant dye, RhB. A remarkable degradation efficiency of 98% within 200 min was achieved under sunlight irradiation, and a degradation constant of 0.017 min^−1^ was obtained (Varadavenkatesan et al., 2019).

ZnO nanoflowers were obtained using *Bridelia retusa* leaf extract. Within a span of 165 min, under solar irradiation, ZnO NPs showed the photocatalytic degradation of RhB dye up to 94.74%. Exhibiting pseudo-first-order kinetics, the process had a degradation constant of 0.0109 min^−1^ (Vinayagam et al., 2021).

Karhik et al. reported the development of Cu-doped ZnO NPs from *Synadium grantii* plant extract (Karthik et al., 2022). The photocatalytic studies of the NPs were studied using MB, Indigo Carmine (IC), and RhB organic pollutants. 3% and 5% Cu-doped samples exhibited a superior photocatalytic activity for MB, IC, and RhB dyes.

Ag/ZnO nanocomposites were prepared using *Urtica dioica* leaf extract (Sohrabnezhad and Seifi, 2016). Their photocatalytic activity was assessed on MB.

Synthesis of nickel oxide (NiO), CuO, and ZnO was carried out by the solution combustion method, making use of *Centella asiatica* extract as a fuel/reductant (Alam et al., 2021). ZnO exhibited a greater photocatalytic activity than NiO and CuO on acid red 88 (AR88).

Cauliflower-shaped ZnO NPs were developed using *Alchemilla vulgaris* (Rajendrachari et al., 2021). The band gap of ZnO NPs was studied by UV–vis spectroscopy via Tauc’s method, and it was determined to be 3.27 eV. The photocatalytic activity of ZnO NPs was executed against RhB under the illumination of an AM 1.5 solar simulator.

ZnO- and manganese (Mn)-doped ZnO NPs using the seed extract of *Cassia angustifolia* were synthesized (Dhivya et al., 2022). The effective photocatalytic activity of ZnO NPs and Mn-doped ZnO achieved 84 and 95% decolorations of water pollutant MB dye, respectively, with H_2_O_2_ and UV radiation by the changing parameters like dye concentration, amount of catalyst, and pH.

Reduced graphene oxide-zinc oxide (RGO-ZnO) NPs were prepared using *basilicum* leaves extract (Raouf Malik et al., 2022). In photocatalytic activity, the ZnO NPs and RGO-ZnO NPs were tested as the catalyst and degraded the RhB dye 91.4% and 96.7% under UV–vis light. RGO-ZnO showed better results in photocatalytic activity against the pure ZnO NPs.

Binary metal oxide zinc oxide-cobalt oxide (ZnO-Co_3_O_4_) nanocomposites were prepared by the sol–gel method using the organic compounds of *Abies pindrow* plant leaves (Shaheen et al., 2022). The ZnO-Co_3_O_4_ catalyst exhibited excellent catalytic potential to degrade MO with 73% and 99% degradation efficiencies under dark and light conditions, respectively, within 15 min.

Ag-doped ZnO nanowires were developed using the medicinal plant *Pedalium murex* (Mangala Nagasundari et al., 2021). The NPs exhibited a photocatalytic degradation of about 92.43% of MB dye in 150 min under UV irradiation.

Biosynthesis of ZnO NPs making use of *Sonneratia alba* has been reported (Khan et al., 2022). The as-synthesized ZnO NPs showed excellent photocatalytic activity against Ethidium bromide (EtBr) and Brilliant green (BG) dye. Kinetic studies showed degradation efficiency toward EtBr (97.07%) and BG (95.01%).

SnO_2_-ZnO heterojunction nanocomposites were prepared using *Urtica dioica* leaf extract (Ebrahimian et al., 2021). The photocatalytic activity of the sample was investigated for the degradation of RhB under UV irradiation. According to the results, the RhB degradation efficiency was obtained to be 91.9% within 55 min, which can be compared with 91.7% at 190 min and 85% at 140 min over SnO_2_ and ZnO, respectively.

Cu-doped NiO NPs were synthesized by the Okra plant extract. XRD results showed that the size of NPs under optimal conditions (at the temperature of 400°C and % 3 Cu-doped) was 10.66 nm with a face-centered cubic structure (Ghazal et al., 2021). Photocatalytic activity of these NPs on MB, studied under UV light indicated 78% of degradation throughout 105 min.

The extract of the medicinal plant *Tribulus terrestris* was employed to synthesize NiO NPs (Khan et al., 2021). The ecotoxicity of CR and components derived from the dye was investigated using the Ecological Structure Activity Relationship (ECOSAR) program (Khan et al., 2021).

Ni/NiO NPs were prepared in an eco-friendly way using the seed extract of *Lactuca Serriola* (Ali et al., 2022). These NPs exhibited very good photocatalytic activity on CV.

Gold-titanium dioxide (Au-TiO2)-NPs were synthesized by the green method using *Avverhoa bilimbi* and *Pandanus amaryllifolius*. MB was degraded 80% by Au-TiO_2_-NPs, and the reaction followed the second order (Yulizar et al., 2019).

*Acorus calamus* leaf extract was used to develop TiO_2_ NPs (Ansari et al., 2022). The dye degradation activity of the synthesized NPs suggested that TiO_2_ NPs were more involved in RhB degradation than conventional hydrogen peroxide, which needs the use of a catalyst. Under visible light irradiation, the increased photocatalytic action of the biosynthesized TiO_2_ NPs was responsible for the degradation efficiency of 96.59% of the RhB.

*Cedrous deodara* extract was used to synthesize Cu@TiO_2_ NPs (Ramzan et al., 2021). Under sunlight irradiation, Cu@TiO_2_ NPs showed 95% of MB; on the other hand, it showed 73% dye degradation under visible light irradiation. The difference of dye degradation was because of the active participation shown by e^−^ and h^+^ separations, which resulted in slowing the recombination rate of electron hole pairs as well.

Lemon peel extract was employed to synthesize TiO_2_ NPs for the first time (Nabi et al., 2022). The Tauc plot prepared from the UV–vis spectrum showed a band gap of 3.08 eV. The particles were spherical anatase crystals with the size 80–140 nm. The particles exhibited more than 70% photocatalytic activity on RhB.

*Pulicaria undulata* extract-mediated eco-friendly synthesis of TiO_2_ has been reported by Khaleel Al-hamoud et al (Al-hamoud et al., 2022). They were tested for the degradation of MB and MO under UV–vis light irradiation. Due to the small size and high dispersion of NPs, almost complete degradation (∼95%) was achieved in a short period of time (between 1 and 2 h). No significant difference in the photocatalytic activity of the TiO_2_ NPs was observed even after repeated use (three times) of the photocatalyst.

Magnesium oxide (MgO) NPs were developed using *Texas sage* plant extract (Ahmad et al., 2022). A characteristic metal and oxygen binding peak was observed at 296 nm in the FTIR spectrum confirmed the Mg-O formation. The 90% of degradation efficiency against MB was observed within 120 min of visible light irradiation (Ahmad et al., 2022).

Cobalt-doped cerium oxide (Co-CeO_2_) NPs were prepared in a media containing *Salvadoral persica* extract. The photocatalytic activity of Co-CeO_2_ NPs was tested on the AO7 dye, and it was observed that the amount of Co. doped CeO_2_ can be effective on the photocatalyst performance. The best photocatalytic performance was observed and recorded in the case of 7% Co. doped CeO_2_ NPs (Hamidian et al., 2021).

CeO_2_ NPs were prepared using banana peel. SEM results showed that the size of synthesized NPs was in the range of 4–13 nm (Miri et al., 2021). The experiment of photocatalytic activity showed that the synthesized NPs can remove 81.7% of AO7 in 180 min under visible light.

Green synthesis of ceria (CeO_2_) by *Phoenix dactylifera* (Ajwa dates), *Coriandrum sativum* (Coriander), *Ocimum tenuiflorum* (Black tulsi), *Camellia sinensis* (green tea), and *Hibiscus* flower has been reported (Iqbal et al., 2021). The photocatalytic activity of various green synthesized nanoceria for the photodegradation of amido black dye was investigated, and the optimum degradation was obtained for *Phoenix dactylifera* synthesized nanoceria.

Kannan et al. have reported the synthesis of zinc sulfide (ZnS) NPs using extracts of *Acalypha indica* and *Tridax procumbens* (Kannan et al., 2020)*.* The samples were found to have excellent photodegradation efficiency for MB dye. From this investigation, 1.0 mg of ZnS-40 mL NPs exhibited an excellent MB dye degradation efficiency (98%) under 180 min of the visible light irradiation.

Au NPs were developed by green synthesis using green tea leaf extract as a reducing and stabilizing agent (Singh et al., 2021). These Au NPs were studied to understand their catalytic role in the photocatalytic degradation of MB under daylight conditions.

Tungsten oxide (WO_3_) NPs were prepared hydrothermally by basil leaves extract (Bayahia, 2022). WO_3_ NPs were examined for their photocatalytic activity on MB.

Tin tungstate (SnWO_4_)/ZnO nanocomposites were prepared using *Muntingia calabura* leaf extract (Elviera et al., 2022). Results of the photocatalytic experiment using MB as a model pollutant showed good results.

Copper iodide (CuI) NPs were synthesized using *Hibiscus rosa-sinensis* flower extract (Archana et al., 2022). Batch adsorption studies of Reactive Red 256 (RR) and Reactive Black 5 (RBL) were probed in the presence of CuI, and the respective adsorption capacity of RR and RBL were found to be 135.22 and 176.42 mg/g.

Biosynthesis of zirconium oxide (ZrO_2_) NPs was carried out using *Wrightia tinctoria* leaf extract (Al-Zaqri et al., 2021). The photocatalytic degradation of organic dye Reactive Yellow (RY 160) under sunlight irradiation using biosynthesized ZrO_2_-NPs was performed. The biosynthesized ZrO_2_-NPs exhibited 94% degradation for RY 160 dye for 120 min.

Platinum (Pt) NPs were synthesized by using *Salix Tetraspeama* leaf extract (Ramachandiran et al., 2021). The effective parameters on the Pt catalytic reduction were fully optimized under MB with the higher degradation efficiency being observed at 90%.

NiO nanosticks were synthesized with the aid of graviola leaf extract (Rajan et al., 2017). These nanosticks showed excellent photocatalytic activity on Rose Bengal dye (Rajan et al., 2017).

Cobalt ferrite (CoFe_2_O_4_) NPs were prepared using the aqueous solution of *torajabin* (Miri et al., 2022). The photocatalytic degradation activity of CoFe_2_O_4_ NPs was assessed on Acid Orange 7 (AO7) dye under simulated sunlight exposure where a high level of photodegradation was displayed by the NPs.

*Ocimum tenuiflorum* assisted green synthesis of CuO NPs has been reported (Sharma et al., 2021). Studies on photocatalytic activity indicated that CuO NPs could be employed as a photocatalyst in the degradation process MO. Up to 96.4 ± 0.83% degradation of MO was obtained in just 24 min.

The above examples very clearly indicate that various metals, metal oxide NPs, and composites could be obtained from green synthetic approaches that can be used for wastewater treatment. Distinctive methods are available to remove the organic dye contaminants from wastewater, including filtration, ion exchange, coagulation, aerobic/anaerobic decomposition, ozone treatment, and photocatalytic processes. Among these methods, nanoparticle photocatalysts show great potential as an alternative to physical, chemical, and biological methods. In addition, nanocatalysts are inexpensive, chemically stable, environmentally friendly, and rapidly oxidizable.

### Photocatalytic Degradation of Pesticides and Oil by NPs Developed by Green Approaches

Heavy metals, fertilizers, and pesticides used in agriculture have lowered the availability of clear water for drinking and crop irrigation. Nanotechnology can play an essential role in ensuring the quality and purity of a long-term productivity system. Pesticide levels in water have risen as a result of their widespread use in contemporary agriculture. Pesticides have been shown in several studies to be hazardous to individuals and the environment. The type of pesticide and the performance of the treatment process influence which water treatment method is best for pesticide removal (Rasheed et al., 2019b).

One of the issues in water resource management is oil-contaminated water. To comply with the environmental authorities’ discharge requirements, it is critical to remove oil droplets from water. Apart from removing dyes from contaminated water, green synthesized NPs can be employed to remove pesticides and oils and they were found to be very effective.

Synthesis of flower-shaped CuO and NiO was carried out, making use of *Capparis decidua* leaf extract. These NPs were employed for the photocatalytic degradation of Lambda-cyhalothrin (L-CHT) (Iqbal et al., 2021c).

*Aegle marmelos* leaf extract was used to develop NiO NPs by the green approach. They were evaluated for their *in vitro* cytotoxicity against A549 cancer cell line. They were also proved to be an efficient photocatalyst for the degradation of 4-chlorophenol (Angel Ezhilarasi et al., 2018).

Josiane Peternela et al. reported the impregnation of CuO NPs on activated carbon, making use of Pomegranate leaf extract. Atrazine pesticide, diclofenac, caffeine, and nitrate were used as pollutants to test the adsorption capacity of the impregnated carbons (Peternela et al., 2017).

Pijushkanti Purkait et al. reported the synthesis of ZnO NPs using *Trema orientalis* leaf extract. They reported that the rate of photocatalytic degradation of zoxamide increased with the increasing concentration of ZnO from 10 mg/L to 50 mg/L (Purkait et al., 2020).

Synthesis of NiO NPs using *Solanum trilobatum* extract has been reported. They were examined for the photocatalytic degradation of 4-chlorophenol (Angel Ezhilarasi et al., 2020).

Papaya peel extract-mediated green synthesis of CuO NPs has been reported (Phang et al., 2021). The sample was found to be crystalline with a band gap energy of 3.3 eV. The photocatalytic activity of the sample was assessed by the photodegradation of Palm oil mill effluent (POME). The results revealed that CuO NPs had a significant photocatalytic performance in degrading POME with reduced phytotoxicity and can thus be used as a promising photocatalyst in POME wastewater treatment (Phang et al., 2021).

Bimetallic metal oxides namely nickel-copper oxide (NiCuO), copper-chromium oxide (CuCr_2_O_4_), and nickel-chromium oxide NPs were developed by the green approach with the aid of *Aegle marmelos* leaf extract. These NPs were evaluated for the removal of various harmful phenols (phenol, 2, 4-dinitrophenol, and 3-aminophenol) from simulated water (Rani and Shanker, 2018).

Graphene oxide–Ag nanoparticle composites were developed with the aid of *Cucurbita maxima* leaves extract. Photocatalytic degradation of Chlorpyrifos [O, O-diethy O-(3,5,6 trcholr-2-pyridyl) phosphorothioate)] was carried out with the produced graphene oxide–Ag NPs. (Chinnappa et al., 2022).

The photocatalytic activity of various green synthesized NPs against different dyes, pesticides, and oils is presented in Table 2.

**TABLE 2:** Photocatalytic activity by green synthesized NPs.

Entry	Nanoparticles	Plant	Dye/Pesticide/Oil	References
1	Ag	*Bryophyllum pinnatum*	MB and RhB	Chandraker et al., (2021a)
2	Ag	*Ageratum conyzoides*	MB	Chandraker et al., (2019)
3	Ag	*Zanthoxylum armatum*	Safranine O, MR, MO, and MB	Jyoti and Singh, (2016)
4	Ag	*Sonchus arvensis*	MB	Chandraker et al., (2019)
5	Ag	*Onion, Tomato, Acacua catechu*	MO, MR, CR	Chand et al., (2020)
6	Ag	*Kalanchoe diagremontiana*	MB	Molina et al., (2019)
7	Ag	*Shorea robusta*	RhB	Shaikh et al., (2020)
8	Ag	*Calotropis procera*	MO	Chandhru et al., (2021)
9	Ag	*Cordia dichotoma*	MO	Dave et al., (2021)
10	Ag	*Terminalia bellirica*	MB	Sharma (2021)
11	Ag	*Berberis vulgaris*	MO	Hashemi et al., (2022a)
12	Ag	*Magnifera indica*	MB	Panneerselvi et al., (2022)
13	Ag	*Catharanthus roseus*	MO	Muthu et al., (2021)
14	Ag	*Sambucus ebulus*	MO	Hashemi et al., (2022b)
15	Ag/AgCl	*Cissus quadrangularis*	MO	Priyadarshini et al., (2021)
16	Ag/AgCl	*Azadiracta indica*	MB	Panchal et al., (2021)
17	Ag/ZnO	*Urtica dioica*	MB	Sohrabnezhad and Seifi, (2016)
18	Ag doped ZnO	*Pedolium murex*	MB	Mangala Nagasundari et al., (2021)
19	Graphene oxide-Ag	*Crucubita maxima*	Chlorpyrifos	Chinnappa et al., (2022)
20	Cu	*Jatropha curcas*	MB	Ghosh et al., (2020b)
21	Cu	*Ageratum houstonianum Mill*	CR	Chandraker et al., (2020)
22	Cu	*Celastrus peniculatus willd*	MB	Mali et al., (2020)
23	CuO	*Ruellia tuberosa*	CV	Vasantharaj et al., (2019a)
24	CuO	*Abutilon indicum*	AB 210	Ijaz et al., (2017)
25	CuO	*Psidium guajava*	NB, RY 160	Singh et al., (2019b)
26	CuO	*Rubia cordifolia*	MB, CV	Vinothkanna et al., (2022)
27	CuO	*Pouteria compechiana*	MB	Navada et al., (2021)
28	CuO	*Cuperus pangorei*	RhB	Parvathiraja and Shailajha, (2021)
29	CuO	*Ocimum tenuiflorum*	MO	Sharma et al., (2021)
30	CuO	*Papaya*	POME	Phang et al., (2021)
31	CuO, Ag, MnO_2_	*Kalopanax pictus*	CR	Moon et al., (2018)
32	CuO NPs on Carbon	*Pomegranate*	Atrazine	Peternela et al., (2017)
33	ZnO	*Panous*	MB, EY, MG	Kaliraj et al., (2019)
34	ZnO	*Plectranthus amboinicus*	MR	Fu and Fu, (2015)
35	ZnO	*Cyanometra ramiflora*	RhB	Varadavenkatesan et al., (2019)
36	ZnO	*Bridelia retusa*	RhB	Vinayagam et al., (2021)
37	ZnO	*Sonneratia alba*	BG	Khan et al., (2022)
38	ZnO	*Alchemilla vulgaris*	RhB	Rajendrachari et al., (2021)
39	ZnO	*Trema orientalis*	Zoxamide	Purkait et al., (2020)
40	Cu doped ZnO	*Synadium grantii*	MB	Karthik et al., (2022)
41	ZnO, Mn doped ZnO	*Caesia angustifolia*	MB	Dhivya et al., (2022)
42	RGO-ZnO	*Ocimum basilicum*	RhB	Raouf Malik et al., (2022)
43	SnO_2_-ZnO	*Aloe vera*	MO	Sudhaparimala and Vaishnavi, (2016)
44	SnO_2_-ZnO	*Urtica dioica*	RhB	Ebrahimian et al., (2021)
45	ZnO-Co_3_O_4_	*Abies pindrow royle*	MO	Shaheen et al., (2022)
46	SnWO_4_/ZnO	*Muntingia calabura*	MB	Elviera et al., (2022)
47	NiO	*Tribulus terrestris*	CR	Khan et al., (2021)
48	Ni/NiO	*Lactuca serriola*	CV	Ali et al., (2022)
49	NiO	*Graviola*	MO	Rajan et al., (2017)
50	NiO	*Aegle marmelos*	4-CHP	Angel Ezhilarasi et al., (2018)
51	NiO	*Solanum trilobatum*	4-CHP	Angel Ezhilarasi et al., (2020)
52	NiO, CuO, ZnO	*Centella asiatica*	AR88	Alam et al., (2021)
53	Cu doped NiO	*Okra*	MB	Ghazal et al., (2021)
54	CuO and NiO	*Capparis decidua*	L-CHT	Iqbal et al., (2021c)
55	CdS	*Dicliptera Roxburghiana*	MB	Ullah et al., (2021)
56	Cu-Co-Ni	*Origanum vulgare*	MB	Alshehri and Malik, (2020)
57	CoO_2_	*Taraxacum officinale*	Direct yellow-142 and MO	Rasheed et al., (2019a)
58	α-Fe_2_O_3_	*Carica papaya*	Remazol yellow RR dye	Bhuiyan et al., (2020)
59	IO	*Teucrium polium*	MO	Kouhbanani et al., (2019)
60	FO	*Peltophorum pterocarpum*	MB	Anchan et al., (2019)
61	FeO	*Ruellia tuberosa*	CV	Vasantharaj et al., (2019b)
62	Fe-P	*Eucalyptus tereticornis, Melaleuca nesophila, and Rosemarinus officinalis*	AB 194	Wang et al., (2014b)
63	Fe_3_O_4_	*Mentha pulegium, Artemisia herba-alba*	MB, CR	Meneceur et al., (2022)
64	Iron oxide	*Peltophorum pteerocarpum*	RhB	Shah et al., (2022)
65	Pd	*Pimpinella tirupatiensis*	CR	Narasaiah et al., (2017)
66	TiO_2_	*Acorus calamus*	RhB	Ansari et al., (2022)
67	Cu@TiO_2_	*Cedrous deodara*	MB	Ramzan et al., (2021)
68	TiO_2_	*Lemon peel*	RhB	Nabi et al. (2022)
69	TiO_2_	*Pulicaria undulate*	MB, MO	Al-hamoud et al., (2022)
70	Au/TiO_2_	*Averrhoa bilimbi and Pandanus amaryllifolius*	MB	Yulizar et al. (2019)
71	MgO	*Texas sage*	MB	Ahmad et al. (2022)
72	CeO_2_	*Banana peel*	AO7	Miri et al. (2021)
73	CeO_2_	*Phoenix dactylifera, Coriandrum sativum, Ocimum tenuiflorum, Camellia sinensis, and Hibiscus flower*	Amido black	Iqbal et al. (2021)
74	Co-CeO_2_	*Salvadoral persica*	AO7	Hamidian et al. (2021)
75	ZnS	*Acalypha indica, Tridax procumbens*	MB	Kannan et al. (2020)
76	Au	*Tea*	MB	Singh et al. (2021)
77	WO_3_	*Basil*	MB	Bayahia (2022)
78	CuI	*Hibiscus rosa-sinesis*	RR	Archana et al. (2022)
79	ZrO_2_	*Wrightia tinctoria*	RY160	Al-Zaqri et al. (2021)
80	Pt	*Salix tetrasperma*	MB	Ramachandiran et al. (2021)
81	CoFe_2_O_4_	*Torajabin*	AO7	Miri et al. (2022)
82	Ni-CuO, Cu-Cr_2_O_4_, Ni-Cr oxide	*Aegle marmelos*	Phenol, 2,4-dinitrophenol, 3-amino phenol	Rani and Shanker (2018)

## Conclusion and Future Plan

Green synthesis technology is a clean, non-toxic, and environmentally friendly technology for synthesizing metal NPs and is of great interest due to its commercial perspective and feasibility. In this review, we document the production of different metal NPs from various plant extracts and their characterization by UV–vis spectroscopy, XRD, FT-IR, SEM, and TEM. When the optical band value is less than 5 eV, it favors the photocatalytic activity. The NPs from plant extracts had a very effective degradation activity on pollutant organic dyes and purification process on the contaminated water. Recent advances in research in metallic NPs suggest that the integration of metallic NPs and semiconductors is a promising strategy in the development of efficient visible light-responsive materials. Research on the challenges that arise in this area will lead to rapid progress in this area. It is reasonable to assume that such efforts will enable obvious breakthroughs in the development of metallic NPs and make them an important strategy in visible light photocatalysts. To date, the photocatalytic efficiency of the green synthetic photocatalysts available is noteworthy. However, their stability, and ease of use are still inadequate. To achieve the photocatalytic sustainability of these photocatalysts, scavengers must be added continuously to improve the photocatalytic efficiency. The lack of industrial application of green synthetic photocatalysts is mainly due to the reasons such as low yields of NPs, workshop setups on a scientifically relevant scale, and low recycling capacity of green synthetic photocatalysts. Thus, protocols need to be modified further for making these methods cost-effective and comparable with traditional methods for the large-scale production of efficient NPs. Improvement of reliable and eco-friendly processes for the synthesis of metallic NPs is a significant step in the field of applied nanotechnology. We believe this review will help new researchers in this field to understand the development of metal NPs in a cleaner way and their applications.
